# Management of Rapunzel Syndrome in a Four-month Pregnant Female with Twins: The First Reported Case?

**DOI:** 10.7759/cureus.3039

**Published:** 2018-07-24

**Authors:** Mohammad Ali Shahab, Ahmed Shahab, Shahab Javid

**Affiliations:** 1 Department of Internal Medicine, Louis A. Weiss Memorial Hospital, Chicago, USA; 2 Department of Urology, Liaquat National Hospital, Karachi, PAK

**Keywords:** bezoar, rapunzel, twin, pregnancy, trichobezoar

## Abstract

A bezoar is a collection of packed indigestible matter that accumulates in the gastrointestinal tract after ingestion by the patient. It may be made of hair (trichobezoar), vegetable or fruit (phytobezoar), or other indigestible materials. Trichobezoars are thought to form due to hair’s natural enduring nature, as they get matted and stick together in the gut. We present the case of a young female who was 16-weeks pregnant with twins presenting to the general surgery clinic with abdominal pain, vomiting and a palpable abdominal mass, which eventually turned out to be a massive trichobezoar manifesting as Rapunzel syndrome. Rapunzel syndrome is a large trichobezoar extending from the stomach into the small intestine. This is perhaps the first reported case of Rapunzel syndrome in a patient pregnant with twins.

## Introduction

A bezoar is a collection of packed indigestible matter that accumulates in the gastrointestinal tract after ingestion by the patient. It may be made of hair (trichobezoar), vegetable or fruit (phytobezoar), milk curd (lactobezoar) or other indigestible materials. Trichobezoars are the most common bezoars and comprise 55% of all bezoars diagnosed [1]. We present a case of trichobezoar in a young female in her second trimester of pregnancy.

## Case presentation

A 25-year-old primigravid female presented to the general surgery outpatient clinic with intermittent epigastric pain, which radiated to the left hypochondrium and left shoulder. This pain was moderate in severity and had persisted for around two months. It was associated with projectile vomiting upon ingestion of solid food materials, as well as watery diarrhea. The patient complained of anorexia and subjective abdominal distension focused in the epigastrium. At that time, the patient had reached her second trimester of pregnancy with no complications in that regard. She reported she was pregnant with twins.

Upon examination, a large nontender mass was palpated in the epigastric region, extending to the left hypochondrium and umbilical region. It was firm in consistency, immobile, and dull to percussion. Auscultation revealed normal bowel sounds. It did not seem to be attached to the overlying skin and the margins could not be palpated superiorly. The rest of her physical examination was unremarkable, with the exception of the expected gravid uterus with twin foetuses.

After further questioning, it became apparent that she had a remote positive history of trichophagia and onychophagia during her childhood. She ate her own hair till the age of 10 and her nails till the age of 17. She had a previous history of iron-deficiency anemia at the age of 18 but had sought treatment with complete resolution.

**Figure 1:**
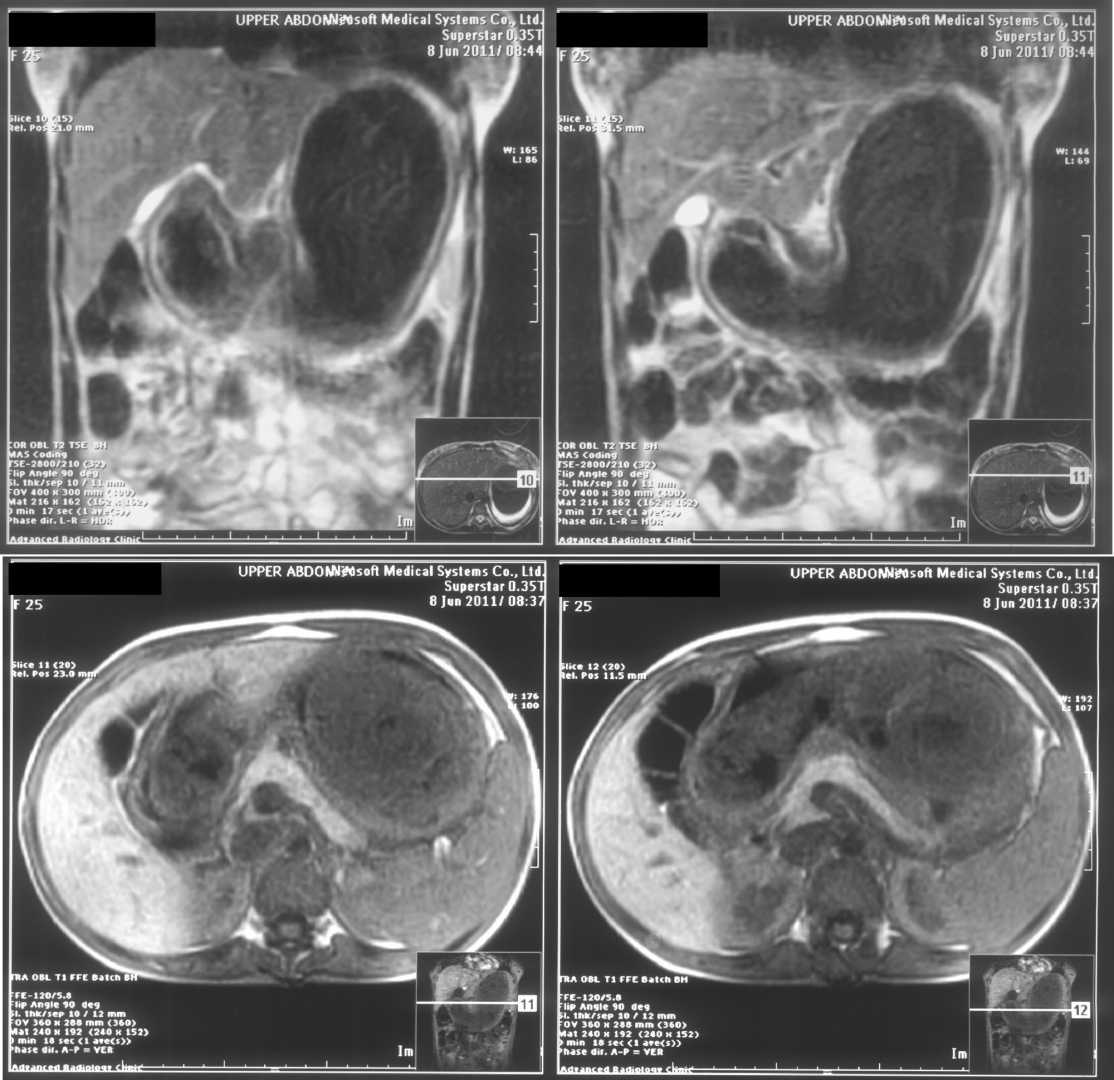
Magnetic resonance imaging (MRI) views showing trichobezoar.

As the mass was large and the extent of it was unknown, abdominal magnetic resonance imaging (MRI) was performed as an initial investigation to explore the provisional diagnosis of a bezoar. The MRI revealed gastric distension with the presence of a soft tissue mass extending throughout the stomach (Figure 1). This confirmed the diagnosis of gastric bezoar, and the patient was admitted as an in-patient and advised for immediate surgery.

**Figure 2:**
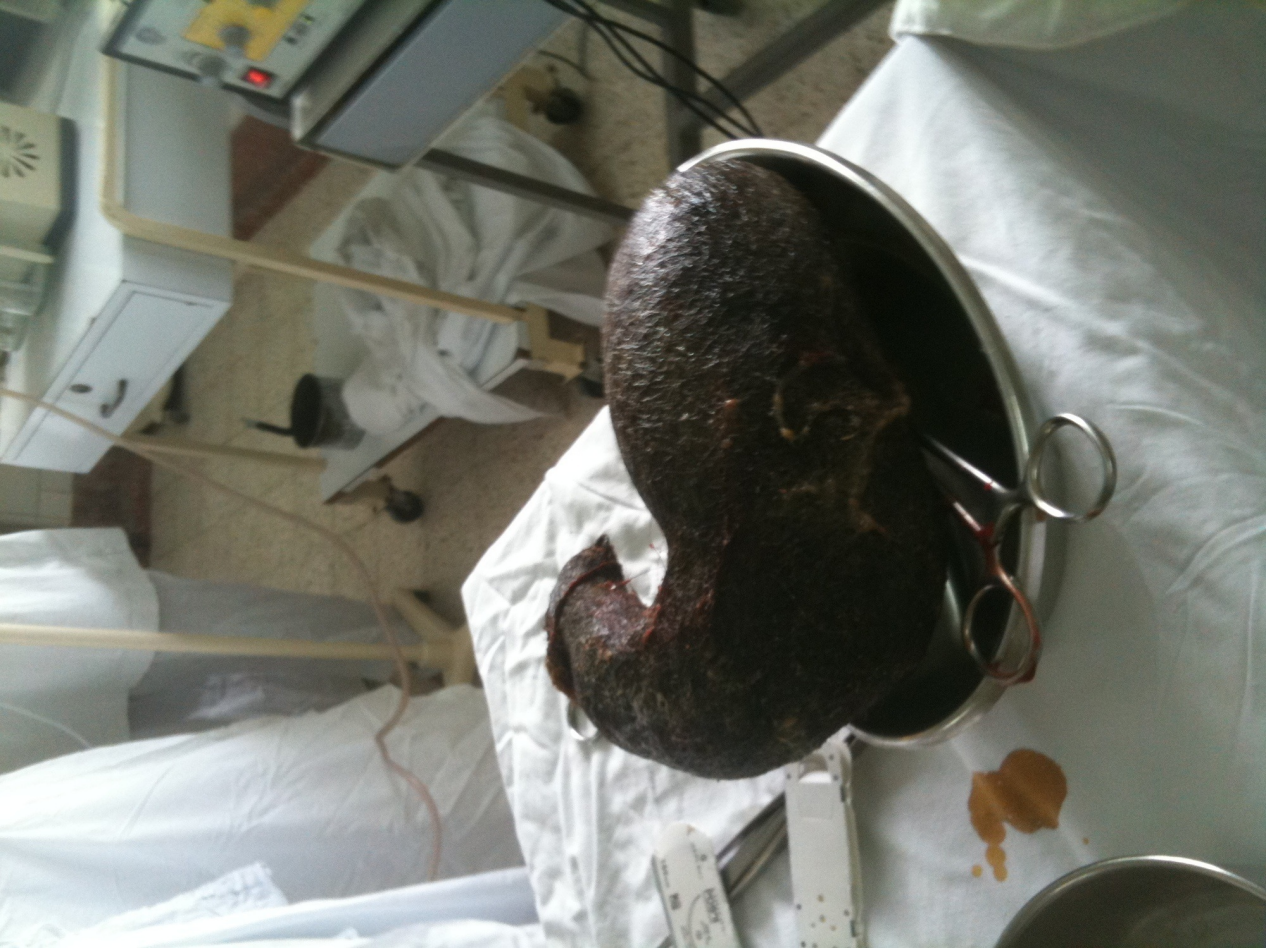
Gross view of removed trichobezoar.

Subsequent to assessment of risk factors and initial blood-work, the patient was scheduled for open laparotomy and surgical bezoar removal five days later. After sterile precautions were taken and appropriate draping was performed, an upper midline incision was made, the muscle layers were retracted, and the stomach was opened between two vicryl sutures. A large trichobezoar was removed, which had taken the exact shape of the distended stomach with a small extension into the duodenum. At the time of removal, its weight was 1.2 kg (Figure 2). The stomach was sutured, and the abdomen and skin were closed in layers. With an unremarkable postoperative stay, the patient was discharged at the sixth postoperative day, with a follow-up visit scheduled in the surgery clinic.

## Discussion

The word bezoar has ancient Persian origin; the original word being ‘padzhar’ meaning protector from poison, was originally assumed to be a stone extracted from an animal’s stomach which could be an antidote for all poisons. Cow bezoars are still used today in modern-day China as a traditional medicine for pain related to cerebral vascular spasm [2]. Today in modern Western medicine ‘bezoar’ refers to any indigestible mass found in the stomach or the intestines. One of the first mentions of bezoar in this modern context was during the 1779 autopsy report by Baudamant of a patient who had died due to gastric perforation and peritonitis [3]. Trichobezoars are thought to form due to hair’s natural slippery and enduring nature. Hair gets trapped in the folds of the stomach lining, resists degradation, and begins to stick to other hair fibers present in the stomach. Eventually, as more hair is ingested, there is enough hair to form a ball-like shape, or bezoar.

As in our patient, it has been reported that 80% of trichobezoars are found in patients younger than 30 years of age, and 90% occur in women [4]. Most patients with trichobezoars have a concurrent history of trichophagia and may also have trichotillomania. It is to be noted that only 1% of patients suffering from trichotillomania actually develop bezoars [5] while about 50% of patients with trichobezoars suffer from trichotillomania. In suspected trichotillomania patients, a detailed hair-root examination should be performed. The presence or absence of telogen hair type is used to distinguish trichotillomania from other hair disorders.

Our case was not that of a simple bezoar, but of a large trichobezoar extending from the stomach into the small intestine, an occurrence known as Rapunzel syndrome. Rapunzel syndrome is named after the classic Grimms’ Fairy tale in which Rapunzel lets down her hair for her lover. Clinical symptoms of Rapunzel syndrome include pain in abdomen, nausea, vomiting, bloating, early satiety, weight loss; clinical signs include a nontender abdominal mass [6]. Complications include intestinal obstruction, gastric ulceration, and possible intestinal intussusception [7]. Malabsorption-related complications including megaloblastic anaemia, protein-losing enteropathies, and iron-deficiency anemia have also been reported [5]. Our patient had the aforementioned constellation of signs and symptoms, with one of our main considerations for immediate surgery being the significant and persistent vomiting that the patient was suffering from. It was not only functionally debilitating for her, but also there was the resulting poor nutritional status that a twin pregnancy patient should not endure. She also had a remote history of treated iron-deficiency anemia, which may or may not have been related to the trichobezoar. The patient insisted that she had abandoned her habit of trichophagia and onychophagia many years prior to presentation. Ideally, a thorough psychiatric evaluation should be performed on such patients. Our patient was advised a psychiatric consultation, but she was not receptive to the idea and maintained she was mentally healthy.

Our patient’s trichobezoar was discovered when she was 16-weeks pregnant with twins. Rapunzel syndrome is a rare syndrome to begin with, and diagnosis and treatment with a concurrent pregnancy is rarer still. We were unable to find any reported case of Rapunzel syndrome in a twin pregnancy in the literature. To our knowledge, therefore, this is perhaps the first reported case of Rapunzel syndrome manifesting symptoms during a twin pregnancy. However, there have been published reports of Rapunzel syndrome in singleton pregnancies [8]. 

Diagnostic investigation for a trichobezoar is endoscopy, which was not possible in our patient due to the size. An ultrasound, computed tomography (CT), or MRI can also be performed. In order to avoid radiation and gain maximal information, we opted for MRI. A trichobezoar can be treated endoscopically, laparoscopically, or via laparotomy. In a study conducted in Amsterdam, four in-hospital cases and 108 previously published case reports of children <15 years, were scrutinized to judge the best treatment option for a trichobezoar. It concluded that laparotomy was the treatment of choice in children and for Rapunzel syndrome it was the only option to be considered [9]. Treatment of choice in pregnant women was discussed in a case report from New Zealand, where a trichobezoar was removed laparoscopically from a woman in her second trimester [10]. The study stated that women undergoing either laparotomy or laparoscopy had an increased risk of low birth-weight babies and premature babies, but no difference in still birth or neonatal death rate compared to nonoperated women. They concluded that surgery was safe in pregnant women [10]. In our case, the logical choice due to the size of the bezoar was laparotomy.

## Conclusions

Rapunzel syndrome is a rarity; we were unable to find more than 30 published cases in the literature, with only four pregnant patients, and indeed none in patients pregnant with twins. We hope that this case brings valuable insight to surgeons and obstetricians encountering a pregnant patient with recurrent and persistent abdominal pain with a concurrent epigastric mass. We recommend having the reasonable suspicion of Rapunzel syndrome or bezoar in such a patient.
